# Investigating the Effect of Artificial Flavours and External Information on Consumer Liking of Apples

**DOI:** 10.3390/molecules24234306

**Published:** 2019-11-26

**Authors:** Isabella Endrizzi, Eugenio Aprea, Emanuela Betta, Mathilde Charles, Jessica Zambanini, Flavia Gasperi

**Affiliations:** 1 Department of Food Quality and Nutrition, Research and Innovation Centre, Fondazione Edmund Mach (FEM), Via E. Mach 1, 38010 San Michele all’Adige, Italy; eugenio.aprea@fmach.it (E.A.); emanuela.betta@fmach.it (E.B.); jessica.zambanini@fmach.it (J.Z.); flavia.gasperi@fmach.it (F.G.); 2 Center Agriculture Food Environment University of Trento/Fondazione Edmund Mach, via E. Mach 1, 38010 San Michele all’Adige, Italy; 3 Sensory and Behaviour Sciences research group, Sportslab, Decathlon SA, 59665 Villeneuve d’Ascq, France; mathildeccharles@gmail.com

**Keywords:** flavouring treatment, SPME/GC-MS, triangle test, sensory profile, apple acceptability, external information

## Abstract

In this paper, the influence of flavour modification, artificially induced, on consumer acceptability of apple fruit is studied. The method consists of modifying the flavour of a real food matrix dipping apples into flavour solutions. Two flavouring compounds (linalool and anethole) that were responsible of “floral” and “anise” aroma descriptors, respectively, were considered here. The effectiveness of flavouring treatments was confirmed by instrumental analysis of volatile compounds profile using solid-phase microextraction gas chromatography/mass spectrometry (SPME/GC-MS) and by discriminative and descriptive sensory analyses. The effect of flavour-impact was evaluated in an informed test on the two flavoured ‘Fuji’ apples: the consumers were asked to evaluate the global liking of the treated and non-treated apples with information regarding the aromatic features. Participants’ additional data on the characteristics on their “ideal apple”, attitudes toward natural food, food neophobia, and demographic data were also recorded by specific questionnaires. A statistically significant effect on liking was found for the flavour factor, whereas external information only affected apple acceptance for subgroups of consumers, depending on their attitude towards food.

## 1. Introduction

Several authors investigated the influence of sensory attributes, both being related to texture and flavour, on consumer liking of apples. For example, the positive correlation between liking and texture parameters like crispness, hardness or juiciness has been demonstrated [1,2,3]. Symoneaux and colleagues [4] reported that crunchiness and sweetness are the main sensory preference key drivers in apple. Endrizzi and colleagues also found that an increase in some texture parameters has less influence on the sweetest apples and a greater influence on the least sweet [5]. The papers reported in the literature suggest that consumers classify apples according to a texture dimension (soft to firm) and a taste dimension (sweet to acid), which suggests that there are two main apple consumer categories: those who prefer firm, juicy, and quite acid apples, and those who like sweeter, but less firm, apples [6,7,8,9,10].

### 1.1. Studying Flavour in Apples

It seems that apple flavour, as the retro-nasal perception of volatile compounds, is a factor of secondary importance for consumers or important just for a limited number of consumers [11,12]. This could be because crunchiness, juiciness, and the ratio between acid and sweet are effectively dominant characters in apples; in other words, these characteristics stimulate our senses first when we eat an apple [13]. Consumer experience when tasting an apple is the result of the multisensory integration of olfactory, taste, and tactile sensations, being modulated by the dynamic evolution of the tasting event and the location of sensory stimuli in the mouth [14]. For these reasons, consumers are commonly not educated to distinguish between taste and flavour and, therefore, their definition of sweet or acid actually includes a set of gustatory and olfactory characteristics. However, the acceptability test results also depend strongly on the variety of apples included in comparison during the study, and thus the selection of samples plays a fundamental role [5,12]. Sometimes, fruits that are developed during breeding programs, with positive flavour attribute, have to be excluded from a study, because available fruits are not enough to conducts the tests (generally one to three trees are available) or because they are considered to be unpleasant for other sensory aspects and cannot be proposed to the consumer [15]. Nevertheless, flavour continues to remain a primary focus in apple breeding [16,17], and it is known that it is an important factor because it also affects other dominant characteristics, such as sweetness [18,19]. In the past, the role of fruit volatiles and tastants on sensory perception has been studied while using model solutions [20], juices [21,22], fruit pulps [23,24], and the injection of flavours into fruit pieces [14,25]. More recently [13], the influence of aroma perception on taste and texture in apple was studied in our laboratory by dipping fruit pieces (cylinders) in a flavour solution.

### 1.2. Studying Intrinsic and Extrinsic Factors

In recent years, the number of publications that investigates consumer choice by simultaneously evaluating intrinsic and extrinsic factors by means of rating-based conjoint experiments has increased (see among others [26,27]). Nevertheless, far fewer studies investigated taste as a factor in a conjoint framework, mainly measuring the effect of different levels of sweetness or sourness, sometimes combined by texture attributes [5,28,29,30,31]. As far as the authors’ know, there are no conjoint studies that examine the effect of different flavours/aromas. It is also known that, external information, as claimed, has a role in consumers’ perception influencing food choices. Several studies have been performed investigating the effect of nutrition and health claims (see among others [5,32,33], information regarding origin [34], production method [35], quality labels [36], or sustainability labels [37]).

### 1.3. Objective of the Study

Here, for the first time as far as the author knows, a conjoint study including tasting was carried out on apple, measuring the effect on liking of different flavours and of external information regarding the flavour of fruit under evaluation. To do that, the effect of flavour-impact was artificially induced in the real food matrix to compare consumer acceptability of unmodified versus flavoured Fuji apples. The effectiveness of the flavouring treatment was evaluated by instrumental and sensory methods. After that, a consumer test was performed to investigate the overall liking of the treated apples. Therefore, the objective of this work is two-fold: to check the applicability and the effectiveness of the flavouring method proposed and evaluate the effect on acceptability of the chosen flavours in apple, in informed conditions.

## 2. Results

The evaluation of the effectiveness of flavouring treatment was achieved by instrumental (Section 2.1.1) and sensory perception verification (Section 2.1.2 and Section 2.1.3). Afterwards, consumers assessed the overall liking of apples (Section 2.2).

### 2.1. Verification of Flavouring Treatment Effectiveness

#### 2.1.1. Quantification of Volatile Organic Compounds

Calibration curves, based on solid-phase microextraction gas chromatography/mass spectrometry (SPME/GC-MS) analysis, were built for the quantification of linalool and anethole present in the apple pulp. The coefficient of variation of the quantification method, which was calculated over 10 consecutive injections, was lower than 14% for linalool and lower than 11% for anethole. Table 1 reports an average amount of flavouring and ethanol penetrating in the apples, as calculated over 10 different fruits per each treatment. Linalool and anethole isomer (estragole) are common constituents that are found in apple headspace [38], generally at low concentration, and they may increase depending on several factors, including ripening and storage. The average amount of linalool and anethole naturally present in the pealed apples used in this study was 0.030 (min 0.026, max 0.031) and 0.019 (min 0.008, max 0.038) mg/kg, respectively. After flavouring treatment, the amount of linalool that was found in the apple pulp was on average 1.05 ± 0.24 mg/kg. Nevertheless, the amount of anethole was 1.02 ± 0.14 mg/kg. The ethanol naturally occurring in the used apples was, on average, 67.1 ± 16.0 mg/kg and only marginally influenced by the soaking treatment (from 52.7 to 81.2 mg/kg). In the Supplementary Materials, we also report the spatial (radial) adsorption trend of linalool and anethole in the apple flesh (Figures S1 and S2). In conclusion, the volatile organic compounds analysis by SPME/GC-MS confirmed that the treatment allowed for the penetration of the flavouring agent into the apple flesh. Furthermore, the two concentrations of flavouring agent employed allowed for obtaining apples with two levels (statistically different) of linalool and anethole.

#### 2.1.2. Triangle Test

Table 2 reports the results of the triangle tests. Apples that were flavoured with anethole, in both concentrations, were perceived to be statistically different from those not treated, while those that were flavoured with linalool were perceived as being statistically different from those that were not treated just in the preparation with the highest concentration. Both compounds were targeted to be presented at the same concentration in the apples after the treatment; nevertheless, the compounds may not elicit the same intensity of aroma at equal concentrations, as confirmed by the data reported in Table 2. We expected a higher rate of correct responses for linalool at low concentration, but the detection thresholds in apple could be different from those found in water, according to the anethole (0.073 mg/L) and linalool (0.00017 mg/L) thresholds in water [39]. At the tested higher concentrations, linalool is more clearly perceived than anethole, perhaps because the floral scent that is given by linalool in apples is recognised as an incongruous sensation, while anethole is not perceived as such: indeed, some varieties are characterised by this attribute in aged apples [40].

#### 2.1.3. Descriptive Sensory Analysis

**Figure 1:**
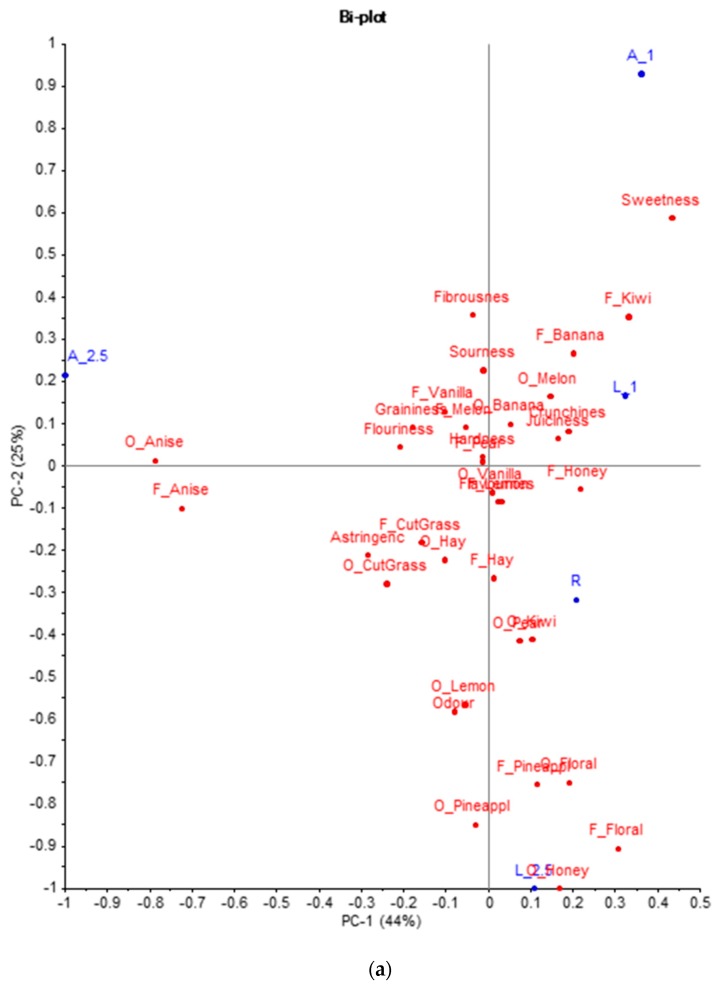

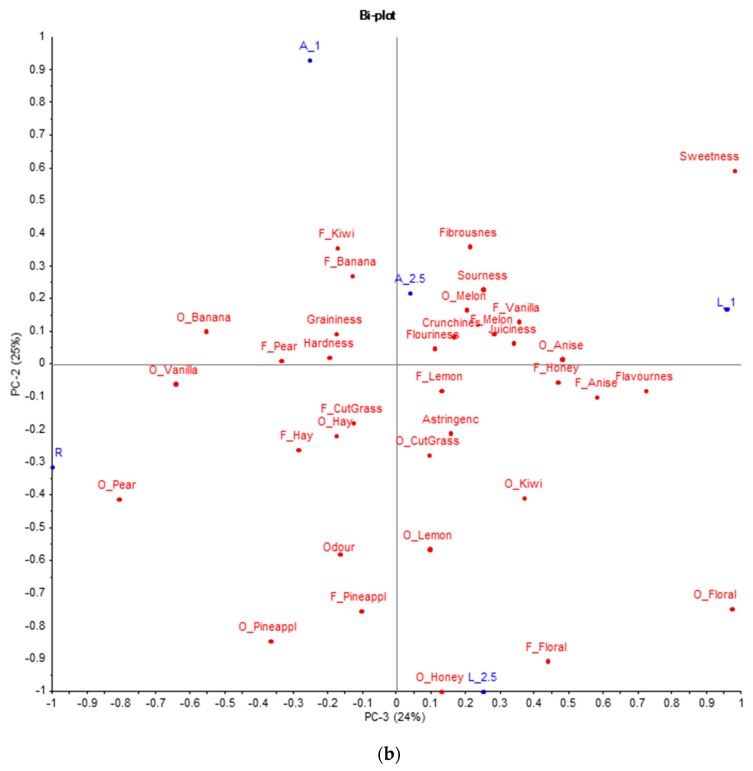
Bi-plot of PCA model on all sensory variables: PC1 vs. PC2 (**a**) and PC3 vs. PC2 (**b**). The six apples, two varieties (Golden Delicious and Fuji) in the three treatments (R, A, and L), are written in bolt.

Descriptive profiling data of the six apples (R, A_1, A_2.5, L_1, and L_2.5) were submitted to PCA to explore similarities and differences among the treated and untreated apples. Figure 1 reports the model on all of the sensory attributes. The analysis revealed that the first three PCs accounted for 93% of the variability. PC1 accounted for 44% of the variability and clearly apple A_2.5 is separated from others along this dimension, whereas sample L_2.5 differs from others along PC2 (25%). Thus, anise flavoured apples are characterised by anise odour and flavour, while apples that are treated with linalool are described with “floral honey” odour, “floral”, and “pineapple” odours and flavours. This is consistent with [25], where fruit pieces that were injected with linalool were mainly described as floral/citrus. In Figure 1b, where PC2 and PC3 (24%) are plotted together, apples that are treated with the solution at the lower concentration (L_1 and A_1) are represented closer and seem to be characterised by higher levels of sweetness. Overall, the results from descriptive sensory analysis revealed that the flavouring method was successful in modifying the samples for their flavour component, but not the other sensory properties.

### 2.2. Consumer Study

#### 2.2.1. Description of Consumer Panels

Fifty-one percent of the panel (*N* = 207) was composed of male, 20% being childless, and 43% living in a family. Seven percent of the panel has a postgraduate degree, 65% was non-smoker, and 64% did sports more than twice a week. For the majority of the 207 consumers an ideal apple should be very crunchy (61%), very juicy (67%), very aromatic (63%), but just fairly acid (92%) and fairly sweet (76%). The panel was segmented in three groups according to its attitude towards natural food interest (NFI): low (31%), medium (38%), and high (31%). A further segmentation on the basis of the food neophobia scale (FNS) was also performed: low (34%), medium (32%), and high food neophobia (34%).

#### 2.2.2. Informed Testing

**Figure 2:**
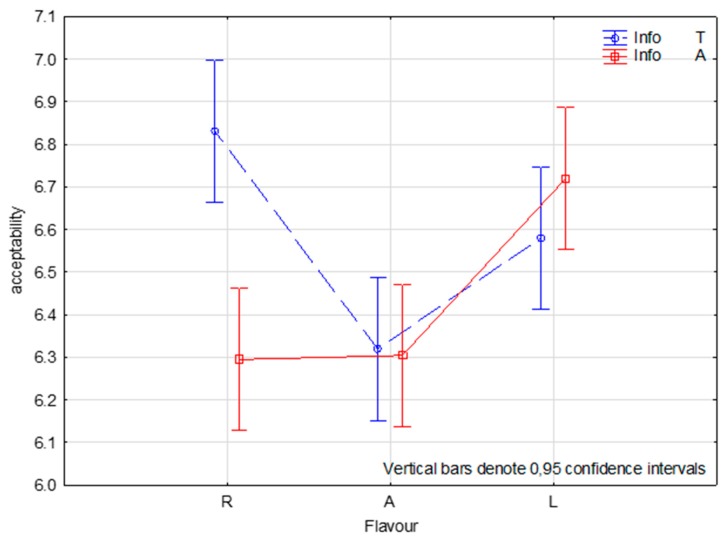
Representation of interaction effect between Flavour (R = reference, A = anethole, L = linalool) and Information (T = traditional, A = chosen for its intense aroma) on liking in the two-way ANOVA mixed effect model.

**Figure 3:**
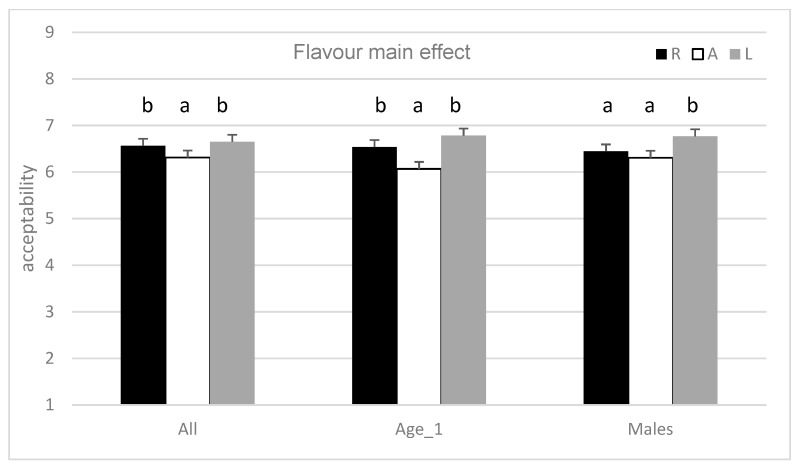
Main effect of flavour (R = reference, A = anethole, L = linalool) on liking of the whole consumer sample (All) and of specific subgroups are reported (Age_1 = 67 consumers under 33 years of age, 105 males). Within the same group, mean values with different letters are significantly different (*p* < 0.05).

**Figure 4:**
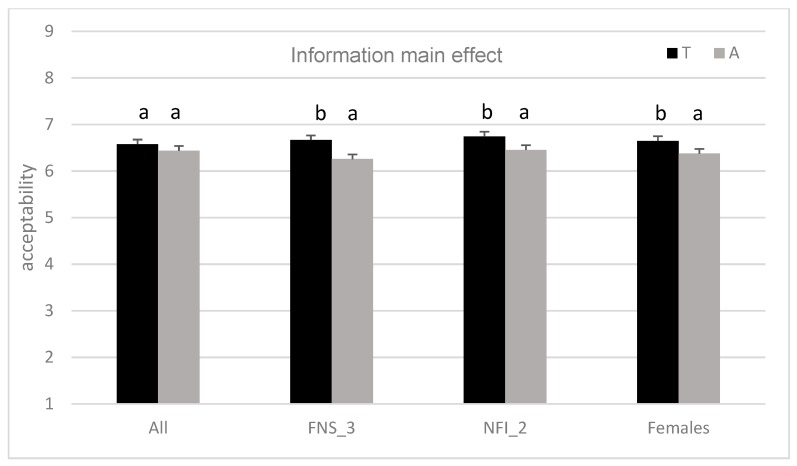
Main effect of information (T = traditional, A = chosen for its intense aroma) on liking of the whole consumer sample (All) and on specific subgroups of it: FNS_3 = 70 consumers with high food neophobia, NFI_2 = 77 consumers with moderate interest towards natural food, 102 consumer females. Within the same group, mean values with different letters are significantly different (*p* < 0.05).

The results of the ANOVA mixed model showed significant main effects on liking for consumer and flavour (*p* < 0.0001 and *p* = 0.007, respectively) and a non-significant main effect for external information (*p* = 0.108). All of the interactions are highly significant (*p* < 0.0001). The interaction between flavour and information (F × I) had the strongest effect (MS = 13.00), followed by the flavour level (MS = 12.77), with anise flavoured apple (A; M = 6.31, SD = 1.64) being the least preferred sample statistically different from floral flavoured apple, which is the most preferred (L; M = 6.65, SD = 1.73). The sample with no added flavour had a different evaluation, depending on submitted information (Figure 2): awarded under the claim ‘traditional’ (R; M = 6.83, SD = 1.58), but penalised if it was labelled as ‘chosen for its intense flavour’ (R; M = 6.29, SD = 1.69). Table 3 reports the results of the ANOVA model (1), recalculated for specific subgroups identified by FNS (FNS_3 = 70 consumers with high food neophobia), NFI (NFI_2 = 77 consumers with moderate interest towards natural food), age (on Age_1 = 67 consumers under 33 years of age), and gender (105 males and 102 females). The significant main effect of flavour is reconfirmed in people under 33 years of age (Age_1) and in males (Figure 3). Additionally, there are significant effects on the liking of external information regarding flavour: females, subjects with a moderate interest towards natural food and subjects with high level of food neophobia evaluated apples that were labelled as ‘traditional’ higher than those claimed as ‘chosen for its intense flavour’ (Figure 4). The finding concerning the F × I interaction was also confirmed in several sub-groups of consumers: males (*p* = 0.011) and females (*p* = 0.016), for those who have a low interest in natural foods (*p* = 0.008) and low neophobia (*p* = 0.015).

## 3. Discussion

### 3.1. Flavouring Method

In the present paper, a new way to investigate flavour impact in apples was proposed. The method used has the advantage of not modifying the apple in its shape, which allows for it to be consumed as a whole fruit if necessary, or in apple slices, as presented here. In previous studies, participants tasted fruit cylinders, cubes, or quartered pieces [13,15,25]. This approach was applied on the Fuji apple, one of the most popular apple varieties in Italy and Europe. It is characterised by appreciated taste and texture attributes with a mild flavour profile [10,41], and this is the reason why we have chosen it. Anethole and linalool have been chosen between the shortlist of those volatile compounds that are already naturally present in apples in low amount [38]. Even if anethole has been shown being noticeable in specific varieties as Ambrosia and in increasing concentration by age [39]. The research was successful in achieving the aim of developing a method that was able to alter the flavour profile of fruit while using apple fruit as an example. The results demonstrate that the compounds reproducibly penetrate into the fruit tissue. This finding is also supported by the perception of the panellists who were able first to discriminate the samples and then to describe them as different in terms of the aromatic profile. The flavouring method should only change the ortho- and retro-olfactory perception and not the other characteristics in order to be effective. This makes sense only from the instrumental point of view, because any different perception that we record could be the result of both a real modification and a multisensory interaction (among the others [42,43]). In our case, the perception of texture attributes remains unchanged between the flavoured samples and the reference, whereas flavoured samples that were prepared with the lowest concentration solution showed a higher level of sweetness (Figure 1). This could be the result of a sensory interaction. It is known that the perception of sweetness in apples is not due only to the sugar content, but also to a strong aromatic component [19]. Here, it seems that when the aroma is perceived clearly and is therefore above the recognition threshold, it is correctly associated and measured; otherwise, it seems to contribute to the evaluation of sweetness. However, further investigations are needed to validate this thesis.

### 3.2. Effect of the Flavour

The goal of this work is to study the impact of flavour on consumer when information is present. Here, the evidence that consumers’ hedonic scores changed in response to the different flavour treatment was demonstrated: the anise-flavoured apple is always the least appreciated, while the floral flavoured is the most appreciated one. The smell of anise is undoubtedly a smell/flavour that divides people according to their liking, even if there is no information regarding that in the literature. Jaeger et al. [25], who injected essences (linalool among the others) into pieces of kiwi, found that their samples that were treated with high linalool were very high in the intensity of floral/citrus attribute and this contributed to diminished acceptability. Indeed, in their study, the sample that was injected with high linalool was the least appreciated. The significant effect of the flavour found for the whole panel (All in Figure 3) is only reconfirmed for male and younger consumers. This is a result that is contrary to the expectations for the authors, who expected mature people and women to be more discriminative because they are generally more open to novelties. Previous works in literature, in fact, described that younger participants showed a higher degree of food neophobia [44] and that women are generally more open than men [45], even if, for gender, some authors described the contrary [46].

### 3.3. Effect of Information

Giving information about “traditional” or “selected flavour” profile while also considering the total mean data did not influence apple acceptance. This result is in accordance with Endrizzi et al. [5], who found that external information are not significant for the whole panel, but rather for specific consumer groups, depending on their sensitivity to the information given. If we associate ‘traditional’ information with the concept of domestic, locally grown, this result is in contrast with those found in the literature where consumers are demonstrated to prefer locally grown apples [47,48,49,50]. This is might be because if, in the cited papers, the concept ‘local’ is always contraposed to ‘non-local’ and thus related to ‘freshness’. Here, we are measuring a different aspect that is more related with flavour profile. However, information regarding ‘traditional apple’ increased the acceptance of apples for specific subgroups of people, depending on consumer characteristics (Figure 4): for females, for highly food neophobic consumers, and for consumers moderately interested towards natural food. These results are not surprising for neophobic consumers who are more comfortable with familiar products and, for females, being generally less neophobic in accordance with the findings that were found by Demattè et al. [44]. With regard to the group of people moderately interested in natural food, as compared to highly NFI people, they evaluate products with reduced or non-fat content as less healthy and those with added sweeteners more healthy [51]. We would have expected the positive effect of traditional information on liking, probably linked to the concept of unmodified, in highly NFI people instead, who consider the importance of eating organic, not processed without additives foods. A further result concerns the interaction between Flavour and Information (F × I), which was found to be significant for the whole panel (Figure 2), but also in the subgroup of consumers with a high interest towards natural food and for the group of neophobics. From this, it emerges that the effect of external information only affects the sample without adding flavour, for which there is, on average, a higher rating if it is presented as ‘traditional’. It seems that the external information only has effect on a product when it is appreciated, penalizing it if presented with the claim ‘chosen for its intense flavour’ since the sample R, the one without added flavour, is undoubtedly the preferred sample. The information does not affect the rating when the product has an unpleasant (unexpected or incongruent) flavour.

## 4. Design and Methods

### 4.1. Flavouring Treatment

#### 4.1.1. Preliminary Trials

The method for modifying the flavour, as already presented by Charles et al. [13] for fruit pieces, consisted here of dipping the whole (unpeeled) fruit in a flavour solution with the objective of modifying the apple flavour with negligible alteration of other sensory attributes. In the preliminary phase of this study, several flavour compounds that were compatible with the apple matrix at different concentration levels were screened and tested (data not presented here). A focus group of researchers, as combined to sensory and instrumental analyses, evaluated the effectiveness of the “dipping” method in relation to the apple variety, the compound used, the concentration to be reached, the impact of other ingredients in the flavouring solution, and to evaluate the congruence between the selected flavour and the apple matrix global perception in order to obtain a realistic product. The focus group started evaluating seven compounds (limonene, cinnamaldehyde linalool, eugenol, anethole, butyl acetate, and benzaldehyde). Just for few of them, the “dipping” method produced a perceptible change in the flavour profile of the apples. Among these, those that induced a realistic flavour in the apples were chosen.

#### 4.1.2. The Flavouring Solutions

As a result of the preliminary trials, two odorants at two levels of concentration were chosen to flavour a batch of ‘Fuji’ apple: linalool (L) at 1 and 2.5 g/L responsible for floral flavour and anethole (A) at 1 and 2.5 g/L responsible for anise flavour. The flavouring solutions were prepared dissolving pure food grade linalool (95%, Sigma-Aldrich, St. Louis, MO, USA) and anethole (99%, Sigma-Aldrich) in a 90% ethanol-water solution, respectively. The 90% ethanol-water solution was chosen to favour the complete dissolution of the flavouring agent, since anethole has weak solubility in water.

No flavouring treatment was considered and the same base solution (90% ethanol-water) without any flavouring agent was used for the preparation of the reference sample (R).

#### 4.1.3. Sample Preparation

The Fuji fruits were harvested in 2013 at a commercial maturity from orchards that were located in Trentino region (North of Italy). A batch of 60 fruits that was homogeneous as possible in ripening, size, and without any visible external damage was bought from local retailers.

As the penetration of the flavouring compound could depend on the size of the whole fruit, the fruits were sorted for weight and divided into three groups on the basis of these measures, and then stored at room temperature (18 ± 1 °C) for 24 h prior to the flavouring treatment. The flavouring treatment was then carried out without distinction on the three groups. The fruits were immersed in the flavouring solution for 1 min. and then removed and placed on a drip grid inserted in a 20 L plastic container. The containers were sealed with parafilm M^®^ and stored for 72 h at 20 °C in dark room prior the tests.

According to this protocol, the following five apple samples were obtained: two “floral” samples flavoured by linalool at 1 and 2.5 g/L (L_1 and L_2,5); two “anise” samples flavoured by anethole at 1 and 2.5 g/L (A_1 and A_2,5), and one “reference” sample (R).

The penetration of the flavour compounds in apple flesh was verified and quantified by SPME/GC-MS analysis, being briefly described in the following Section (Section 4.2.1). Two sensory panels performed discrimination analysis and descriptive sensory analysis to verify the differences between reference and treated apples in terms of human perception (Section 4.2.2).

Only the concentration of 2.5 g/L for both flavourings was chosen for the following consumer test based on the results of these analyses.

### 4.2. Verification of Flavouring Treatment Effectiveness

#### 4.2.1. Quantification of Volatile Organic Compounds

Ten whole fruits for each flavouring treatment (L and A) at each concentration (1 and 2.5 g/L) and further ten whole fruits as reference (R) were submitted to quantitative analysis by SPME/GC-MS in order to investigate the effectiveness and the variability of the flavouring method. Seventy grams from the peeled fruits were diced while using a commercial cutter and immediately inserted in a glass vessel where they were mixed with 75 mL of deionised water, 30 g of sodium chloride, 250 mg of ascorbic acid, and 250 mg of citric acid [38]. From the homogenised samples, a 5 g aliquot was inserted into a 20 mL screw cap vial, suitable for volatile analysis, and it was spiked with 50 µL of 2-octanol (2.5 mg/L) used as internal standards. The vials were placed in the thermostated autosampler tray at 4 °C before the HS-SPME/GC-MS analysis. Calibration curves considering seven calibration points (including blank) were built for the quantification of ethanol (from 0 to 120 mg/kg), linalool (from 0 to 1.00 mg/kg), and anethole (from 0 to 1.10 mg/kg), dissolving known concentrations of the chemicals in apple puree. Volatile compounds from the headspace were extracted and then concentrated on a 2 cm Solid Phase Microextration fibre coated with divinylbenzene/carboxen/polydimethylsiloxane 50/30 μm (DBV/CAR/PDMS, Supelco, Bellefonte, PA, USA). The choice of fibre, as well as analysis parameters, was based on previous work [37]. The fibre was exposed to the apple headspace for 30 min. with the samples being equilibrated at 40 °C. Volatile compounds that adsorbed on the SPME fibre were desorbed at 250 °C in the injector port of a GC that was interfaced with a mass detector, which operated in electron ionization mode (EI, internal ionization source; 70 eV) with a scan range from *m*/*z* 35–350 (GC Clarus 500, PerkinElmer, Norwalk CT, USA). Separation was achieved on a HP-Innowax fused-silica capillary column (30 m, 0.32 mm ID, 0.5 µm film thickness; Agilent Technologies, Palo Alto, CA, USA). The GC oven temperature program consisted of 40 °C for 3 min., and then 40–220 °C at 4 °C/min., stable at 220 °C for 1 min., and then 220–250 at 10 °C/min., and finally 250 °C for 1 min. Helium was used as the carrier gas with a constant column flow rate of 1.5 mL/min.

#### 4.2.2. Sensory Analysis

All of the sensory tests were conducted in the FEM Sensory Laboratory compliant to the EN ISO standards 8589 [52], which was equipped with 22 individual booths while using FIZZ 2.46A software (Biosystemes, Couternon, France) to collect the responses.

##### Triangle Test

The possible unspecific sensory differences in treated apples were investigated while using the triangle method [53] to determine whether flavouring treatment induced perceptible sensory differences in apples. Four consecutive triangle tests were organized to compare the floral and anise flavoured apples at two different concentration levels (1 and 2.5 g/L) with the reference apple (R vs. L_1; R vs. L_2,4; R vs. A_1; R vs. A_2,5). Thirty-eight judges, who were employed at FEM (Fondazione Edmund Mach), with previous experience in discriminative or descriptive sensory analysis, were invited to perform the tests. The apple samples were first peeled, cut using an apple-slicer-corer (12 slices), and then dipped in an antioxidant solution (0.2% citric acid, 0.2% ascorbic acid, 0.5% calcium chloride) [41] preliminarily validated by Corollaro et al. [54]. One slice per sample was served in plastic cups that were labelled with three-digit random codes. Sample presentation order was randomised and counterbalanced over the panel. The judges were asked to evaluate by both smelling and tasting the samples under red light and identify the different sample in each of the four triad randomly presented.

##### Descriptive Analysis

A panel of 10 experienced judges performed the sensory profile of apples according to the descriptive analysis method, while using a consensus lexicon that was developed by Corollaro et al. [41]. For the description of specific odours and flavours, the panel used a list of 11 sensory attributes that were classified in four different categories: fruity (O/F Pear, O/F Banana, O/F Lemon, O/F Kiwi, O/F Pineapple, and O/F Melon), vegetal (O/F Cut grass and O/F Hay), spicy (O/F Vanilla and O/F Anise), and floral (O/F Floral Honey) [13]. In this experiment, in which linalool-flavoured samples are evaluated, the judges were allowed to describe sample odours or flavours while using individual extra attributes not contained in the list because just one floral attribute was included in sensory list. These free evaluations were collected in O/F_Floral, as all extra attributes reported were referable to this category. For each sample (R, A_1, A_2.5, L_1, and L_2.5), randomly presented in the test session, eight apple cylinders (1.8 cm diameter, 1.2 cm high each) were cut, dipped in an antioxidant solution, and then served in a plastic cup that was labelled with three-digit numbers and presented in a balanced order over the panel. The samples were evaluated under red light. Refer to Corollaro et al. for further details regarding the selection of the panel, its performance, general lexicon development, and sensory test procedures [41]. For specific odours and flavours attribute sensory definition, evaluation procedure, and references refer to Charles et al. [13].

### 4.3. Consumer Study

The consumer study was performed in the FEM sensory laboratory by 207 consumers who attended the “open door” event for the FEM’s 140th anniversary (51% males; age: M = 41, SD = 14, Min. = 16, Max = 74). All subjects, who were not paid, declared to like apples and voluntarily joined the sensory evaluations. In addition to socio-demographic data, the participants also provided information regarding the characteristics of their ideal apple, choosing one of the three options (very, fairly, or little) for each ideal feature investigated (crunchiness, juiciness, sweetness, acidity, and aroma). They also provide their attitude toward natural food interest [51] and food neophobia scale [55]: the participants rated their degree of agreement with a series of positive and negative statements conveniently translated in Italian while using a nine-point scale rather than the original seven-point scale [5,24].

#### 4.3.1. Conjoint Test

**Figure 5:**
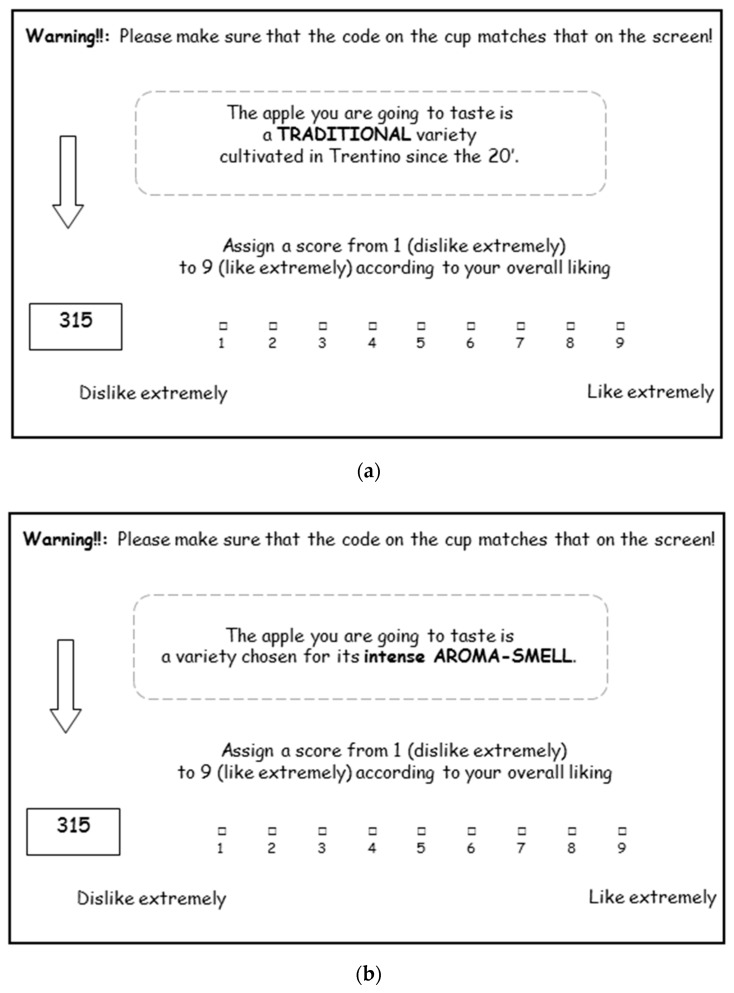
Examples of screen used in the conjoint study: (**a**) the information about traditional apple; and, (**b**) the information about aromatic apple.

The test consisted of an experiment that was evaluated in ‘informed’ conditions combining conjoint analysis with the tasting of the two treated apples (floral and anise flavoured) and the untreated one. Each consumer received six apples in total according to a complete factorial design with three treatments and two information levels: three apples (R, A_2.5 and L_2.5) presented twice every time together with a different claim: ‘traditional’ or ‘chosen for its intense flavour. The claims regarding apple flavour were submitted to consumers on the computer screen (Figure 5) just before tasting the samples. Consumers rated the overall liking of the six apples on a nine-point scale from 1 = “Dislike extremely” to 9 = “Like extremely”. No verbal instructions were given to the consumers prior to testing: the consumers were told to pay attention and carefully read all of the instructions provided during the test.

#### 4.3.2. Sample Preparation

All of the samples were peeled, cut using an apple-slicer-corer (12 slices), dipped in an antioxidant solution, and one slice per sample was served in plastic cups labelled with three-digit random codes and then evaluated under white light. The heaviest apples from G3 (M = 213 ± 6 g, *N* = 84) were submitted to the test first, then those from G2 (M = 197 ± 4 g, *N* = 73), and finally those from G1 (M = 183 ± 5 g, *N* = 50): in this way, each consumer tasted apple slices that were obtained from apples of the same weight range.

### 4.4. Statistical Analyses

For triangle tests, the difference between the two products was considered to be statistically significant when the error was less than or equal to 5% (alpha = 0.05), which corresponded to a level of confidence that was greater than or equal to 95%. Standardised data of descriptive profiling were submitted to PCA (without any weighting option) to explore the similarities and differences among the treated and untreated apples. The liking data that were obtained in the consumer testing were first verified to measure the effect of apple weight (G1, G2, and G3, as mentioned in Section 4.3.2) with a one-way anova on acceptability. The liking data were further analysed using the following model (Equation (1)) with main effects and two-factor interactions for the design variables plus random effect of consumer because no effect was present:

(1) $$y_{\mathrm{ijk}}=\mu +\alpha_{i}+\beta_{j}+C_{k}+{\alpha \beta }_{\mathrm{ij}}+{\alpha C}_{\mathrm{ik}}+{\beta C}_{\mathrm{jk}}+\varepsilon_{\mathrm{ijk}},\;I=1,\;I,\;j=1,\;J,\;k=1,\;K,$$

Here, yijk is the (ijk)th observation, μ is the general mean, αi, βj are the main effects of the two conjoint factors flavour and information, respectively. αβij. is the fixed interaction effect. Ck represents the main effects of consumers, whereas αCik and βCjk are its interaction effects with the design variables, and εijk is the independent random noise. All of these random effects are assumed to be independent and homoscedastic. The same ANOVA model (Equation (1)) was recalculated in specific demographic subgroups of consumers identified by gender (105 males and 102 females), age, natural food interest (NFI), and food neophobia scale (FNS) in order to identify which groups of people were more influenced by the information. According to the 33rd and 66th percentile points, consumers were classified in three groups: for age (Age_1 = 67 consumers under 33 years of age, Age_2 = 70 consumers between 33 and 48 years, and Age_3 = 70 consumers over 48 years), NFI score (NFI_1 = 65 consumers with low interest towards natural food, NFI_2 = 77 consumers with moderate interest, NFI_3 = 65 consumers with high interest), and FNS score (FNS_1 = 71 consumers with low level of food neophobia, FNS_2 = 66 consumers with medium level, and FNS_3 = 70 consumers with high level).

Summary statistics, analyses of variance (ANOVA), post-hoc Tukey’s test, and correlation analysis for the sensory parameters were performed while using Statistica 13.1 (StatSoft, Inc., Tulsa, OK, USA). The Unscrambler X 10.4.1 (CAMO Software AS., Oslo Science Park, Gaustadalléen 21, 0349 Oslo, Norway) was used for the principal component analysis (PCA).

## 5. Conclusions

This study showed that the model system for modifying the flavour of a real structure that is achieved by dipping the whole fruit in flavour solutions is suitable for flavouring the fruits without altering their nature. Overall, tasted apples were acceptable showing scores between 6 and 7. It seems the claim, especially the indication of ‘traditional’, has a positive effect on liking. Different flavouring treatments significantly affect consumers’ preference, depending on their personal liking, while external information only affected apple acceptability for some groups of consumers depending on their food approach. This confirms that flavour is an important factor in apple consumer preference and it could negatively influence the preference as it happened here. External information seems to be less relevant in general, but it can be important for some people to be more receptive to the information provided. The work presented here is an example of how consumer science can contribute to the effectiveness of fruit breeding programs by providing clear consumer advice and a way to early test novel flavour in product development.

## Figures and Tables

**Table 1:** Average amount (mg/kg) and standard deviation (in parenthesis) of ethanol, linalool and anethole in non-flavoured (R) and flavoured (L, A) apples that were treated with two different concentrations (1 and 2.5 g/L), calculated over 10 different fruits per each treatment by solid-phase microextraction gas chromatography/mass spectrometry (SPME/GC-MS).

	R	L_1	L_2.5	A_1	A_2.5
ethanol	67.11 (15.99)	77.16 (25.57)	73.96 (23.91)	81.23 (31.88)	52.68 (17.35)
linalool	0	0.35 (0.07)	1.05 (0.24)	0	0
anethole	0	0	0	0.49 (0.16)	1.02 (0.14)

**Table 2:** Total number of responses, number and percentage of correct responses and the related p-value of each triangle comparison. Statistically significant comparisons (*p*-value < 0.05) are reported in bold.

Comparison	Description	Total Responses	Correct Responses	% of Correct Responses	*p*-Value
1	R vs. L_1	38	18	47	0.051
2	R vs. L_2.5	38	27	71	**<0.001**
3	R vs. A_1	38	22	58	**0.002**
4	R vs. A_2.5	38	19	50	**0.025**

**Table 3:** *p*-values of ANOVA mixed model on the effects of consumer and conjoint factors on liking (All = 207 consumers), on FNS_3 = 70 consumers with high food neophobia, on NFI_2 = 77 consumers with moderate interest towards natural food, on Age_1 = 67 consumers under 33 years of age, on males (105) and females (102). Statistically significant effects (*p*-value < 0.05) are reported in bold.

Source of Variation	Effect	All	FNS_3	NFI_2	Age_1	Males	Females
Consumer (C)	Random	**0.000**	**0.001**	**0.013**	**0.014**	**0.000**	**0.000**
Flavour (F)	Fixed	**0.007**	0.175	0.147	**0.003**	**0.004**	0.098
Info (I)	Fixed	0.108	**0.007**	**0.042**	0.885	0.953	**0.041**
C × F	Random	**0.000**	**0.000**	**0.003**	**0.000**	**0.000**	**0.000**
C × I	Random	**0.000**	**0.006**	0.065	0.210	**0.015**	**0.008**
F × I	Fixed	**0.000**	0.091	0.168	0.063	**0.011**	**0.016**

## Supplementary Materials

The following are available online. Figure S1: Linalool gradient from the peel to the core of the apple after flavouring treatment, Figure S2: Anethole gradient from the peel to the core of the apple after flavouring treatment.
