# Supplementary material for: Investigating the Effect of Artificial Flavours and External Information on Consumer Liking of Apples
Source: Molecules. 2019 Nov 26;24(23):4306. doi: 10.3390/molecules24234306 (PMC6930502; doi:10.3390/molecules24234306)
Supplement: Supplementary file 1 [file molecules-24-04306-s001.zip › molecules-643796-SI.pdf]

molecules

Supplementary Material

Investigating the Effect of Artificial Flavours and External Information on Consumer Liking of Apples

Isabella Endrizzi [1,]* Eugenio Aprea [1,2], Emanuela Betta [1], Mathilde Charles [3], Jessica Zambanini [1], and Flavia Gasperi [1,2]

1 Department of Food Quality and Nutrition, Research and Innovation Centre, Fondazione Edmund Mach (FEM), Via E. Mach 1, 38010 San Michele all'Adige, Italy; eugenio.aprea@fmach.it (E.A.); emanuela.betta@fmach.it (E.B.); jessica.zambanini@fmach.it (J.Z.); flavia.gasperi@fmach.it (F.G.)
2 Center Agriculture Food Environment University of Trento/Fondazione Edmund Mach, via E. Mach 1, 38010 San Michele all'Adige, Italy
3 Sensory and Behaviour Sciences research group, Sportslab, Decathlon SA, 59665 Villeneuve d'Ascq, France; mathildeccharles@gmail.com
* Correspondence: isabella.endrizzi@fmach.it; Tel.: +39-0461-615388

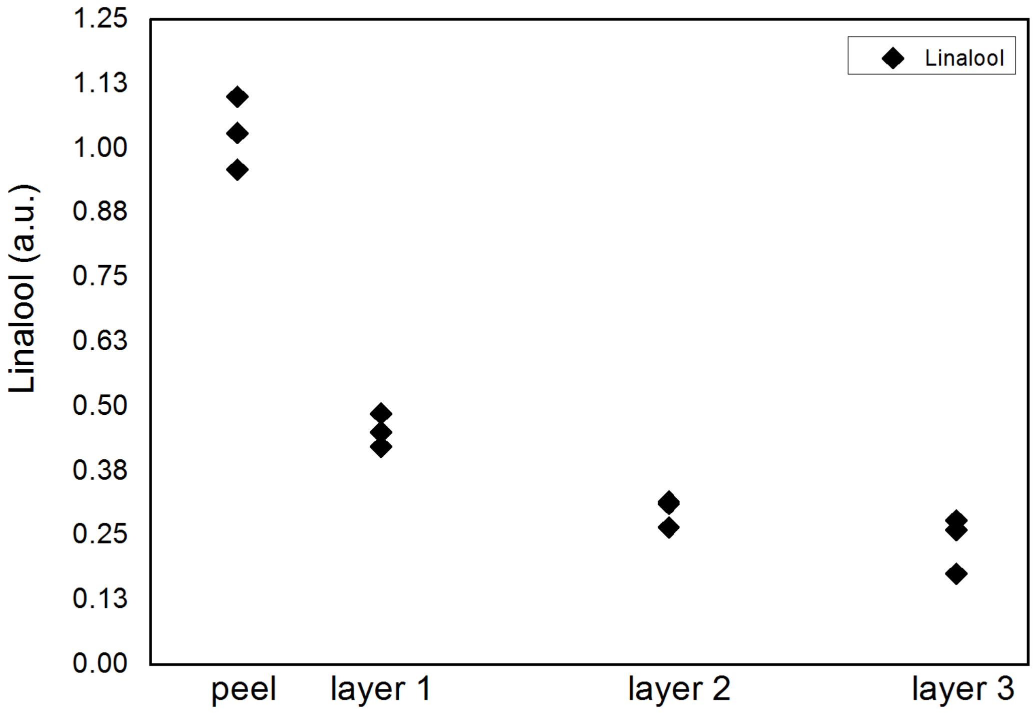

Figure S1. Linalool gradient from the peel to the core of the apple after flavouring treatment. Each layer refers to a region of the fruit pulp of about 1 cm of thickness, where layer 1 is the first under the peel.

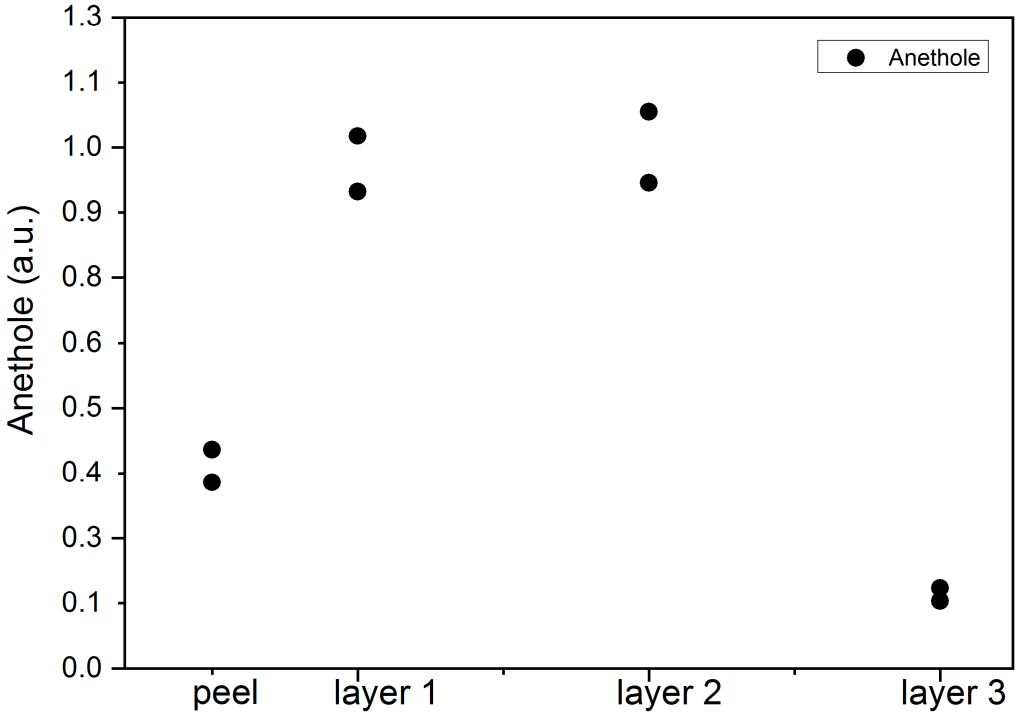

Figure S2. Anethole gradient from the peel to the core of the apple after flavouring treatment. Each layer refers to a region of the fruit pulp of about 1 cm of thickness, where layer 1 is the first under the peel.
